# Clinical application of Al^18^F-NOTA-FAPI PET/CT in diagnosis and TNM staging of pancreatic adenocarcinoma, compared to ^18^F-FDG

**DOI:** 10.1186/s40644-023-00596-1

**Published:** 2023-09-12

**Authors:** Zhehao Lyu, Wei Han, Qi Zhang, Hongyue Zhao, Shan Liu, Yan Wang, Jin He, Changjiu Zhao, Lin Tian, Peng Fu

**Affiliations:** 1 The Department of Nuclear Medicine, the First Affiliated Hospital of Harbin Medical University, Postal Street, Harbin, Heilongjiang Province China; 2 The Department of Radiology, the First Affiliated Hospital of Harbin Medical University, Postal Street, Harbin, Heilongjiang Province China; 3 The Department of Pathology, the First Affiliated Hospital of Harbin Medical University, Postal Street, Harbin, Heilongjiang Province China

**Keywords:** [18F]FDG, Al^18^F-NOTA-FAPI, PET/CT, Pancreatic cancer, Staging

## Abstract

**Purpose:**

This study aimed to investigate the ability of Al^18^F-NOTA-FAPI PET/CT to diagnose pancreatic carcinoma and tumor-associated inflammation with the comparison of ^18^F-FDG PET/CT.

**Methods:**

Prospective analysis of Al^18^F-NOTA-FAPI PET/CT and ^18^F-FDG PET/CT scans of 31 patients from 05/2021 to 05/2022 were analyzed. Al^18^F-NOTA-FAPI imaging was performed in patients who had Ce-CT and FDG PET/CT and the diagnosis was still unclear. Follow-up histopathology or radiographic examination confirmed the findings. Radiotracer uptake, diagnostic performance, and TNM (tumor-node-metastasis) classifications were compared.

**Results:**

A total of 31 patients with pancreatic carcinoma (all were adenocarcinoma) underwent Al^18^F-NOTA-FAPI-04 PET/CT, including 20 male and 11 female patients, with a mean age of 58.2 ± 8.5 years. FAPI-04 PET/CT imaging showed a higher value of SUV_max-15min/30min/60min_, SUV_mean-15min/30min/60min_, TBR_1_, and TBR_2_ in pancreatic carcinoma than FDG (all *P* < 0.01). The mean level of Al^18^F-NOTA FAPI-04 uptake values of the pancreatic ductal adenocarcinoma was higher than that of pancreatitis in both SUV_max-30min_ (*P* < 0.01)_,_ SUV_mean-30min_ (*P* < 0.05), SUV_max-60min_ (*P* < 0.01)_,_ and SUV_mean-60min_ (*P* < 0.01). The FAPI △SUV_max-1_, △SUV_max-2_, and △SUV_mean-2_ uptake values of pancreatic carcinoma were higher than tumor-associated inflammation (all *P* < 0.01). TNM staging of 16/31 patients changed after Al^18^F-NOTA FAPI-04 PET/CT examination with all upstaging changes.

**Conclusion:**

Al^18^F-NOTA-FAPI-04 PET/CT at 15 and 30 min also demonstrated an equivalent detection ability of pancreatic lesion to ^18^F-FDG PET/CT. Delayed-phase Al^18^F-NOTA-FAPI-04 PET/CT can help differentiate pancreatic carcinoma and tumor-associated inflammation. Al^18^F-NOTA FAPI-04 PET/CT also performed better than FDG PET/CT in TNM staging.

**Trial registration:**

Chinese Clinical Trial Registry, ChiCTR2100051406. Registered 23 September 2021, https://www.chictr.org.cn/showproj.html?proj=133033

## Introduction

A 5-year survival rate of 10% for pancreatic ductal adenocarcinoma (PDAC) is a leading cause of cancer mortality worldwide [1]. A comprehensive imaging study of PDAC is essential for accurate initial staging, selection of treatment, and follow-up examinations to detect recurrence and/or metastatic spread. The utilization of ^18^F-FDG PET/CT and PET/MR imaging in clinical contexts is on the rise, however, their usage is limited to the diagnosis of inflammatory and malignant pancreatic ailments [2–4]. A novel PET tracer, fibroblast activation protein inhibitor (FAPI), targets cancer-associated fibroblasts (CAFs) [5]. A CAF in PDAC is derived from pancreatic stellate cells and transforms its tumor-promoting properties by crosstalking with neoplastic cells [6]. The CAF is thought to promote tumor growth, invasion, metastasis, and therapy resistance in PDAC [7, 8]. This study investigated whether Al^18^F-NOTA-FAPI could improve screening efficiency and differentiate between PDAC and tumor-associated inflammation.

## Methods and materials

### Patients

A total of 31 patients who had undergone Al^18^F-NOTA-FAPI-04 PET were prospectively analyzed. All patients gave written informed consent to undergo FAPI PET/CT following the German Pharmaceuticals Act §13(2b) regulations. The clinical translational study of Al^18^F-NOTA-FAPI-04 was approved by the Ethics Committee (approval No. 2021XJSS01) and registered in the Chinese Clinical Trial Registry (ChiCTR2100051406). All patients enrolled in this study signed written informed consent forms. We assessed clinical features, including clinical manifestation, imaging parameters from contrast-enhanced CT (CE-CT), and laboratory panels such as carbohydrate antigen (CA)19–9 and serum amylase. FAPI imaging was performed in patients who had CE-CT and FDG PET/CT and the diagnosis was still unclear. Only patients who had a contraindication (e.g., pregnant women, the start of treatment before FAPI PET/CT examination or patients who refused the procedure) did not undergo Al^18^F-NOTA-FAPI-04 PET/CT imaging. All reported investigations were conducted by the Declaration of Helsinki and with the national regulations. All patients suspected of solid tumors underwent surgery or biopsy to confirm the pathological diagnoses. As defined in previous study [9], it was considered a positive lymph node if the uptake of Al^18^F-NOTA-FAPI-04 or ^18^F-FDG exceeded that of surrounding tissue. For patients suspected of pancreatitis, complementary treatment and imaging follow-up were performed. The minimum follow-up period was three months.

### Radiotracer synthesis

^18^F-FDG was routinely synthesized at the department of Nuclear Medicine of the First Affiliated Hospital of Harbin Medical University, following standard methodology [10]. The synthesis and labeling of Al^18^F‑NOTA‑FAPI-04 have already been described previously [11]. Following the German Pharmaceuticals Act §13(2, b) regulations, indication and labeling of the FAPI-tracers were conducted under the physician's direct responsibility. Injected activities were dependent on labeling yields. According to a previous dosimetry estimate – an effective dose of 1.6 mSv / 100 MBq (2.7 mCi)—an upper limit of 370 MBq (10.0 mCi) regarding radiation exposure, and a lower limit of 100 MBq (2.7 mCi) per exam to achieve a sufficient count rate have been considered [5]. The radiochemical purity was > 95% for both ^18^F-FDG and Al^18^F‑NOTA‑FAPI-04. Al^18^F‑NOTA‑FAPI-04 and ^18^F-FDG tracers met all standard criteria before human administration.

### PET/CT image acquisition

All patients underwent sequential ^18^F-FDG and Al^18^F‑NOTA‑FAPI-04 PET/CT scanning within 1 week. The patients underwent ^18^F-FDG PET/CT examination first. ^18^F-FDG PET/CT was conducted after > 6 h of fasting and among patients with normal blood glucose levels (3.9–11.1mmol/L). The ^18^F-FDG PET/CT was conducted after urinating in quiet, light-avoidance conditions (60min). Al^18^F‑NOTA‑FAPI-04 PET/CT scan was conducted without fasting at 15 min, 30 min, and 1 h, respectively. No patients were required to fast, and venous blood glucose levels were not controlled for Al^18^F‑NOTA‑FAPI-04 PET/CT. Radioactivity ranging from 129.5 to 148 MBq (3.5 to 4.0 mCi) of Al^18^F‑NOTA‑FAPI-04 isotope (Jiangyuan Industrial technology trade Co., LTD, Jiangsu, China, radiochemical purity > 95%) was intravenously injected. The Al^18^F‑NOTA‑FAPI-04 PET/CT were conducted at 15min, 30min and 60min after injection. Both ^18^F-FDG and Al^18^F‑NOTA‑FAPI-04 PET/CT images were acquired using a 16-slice Gemini GXL PET/CT scanner (Philips Medical System). A low-dose CT scan (tube voltage: 120 kV, tube current: 50 mAs, slice thickness: 5.0 mm, pitch: 1.0) was acquired for attenuation correction, and then the PET images were acquired (1.5 min per bed position, 6–7 PET bed positions). According to the agency’s standard clinical protocols, the scan range was from the head to the mid-thigh. The line of response reconstruction algorithm was used to reconstruct the image without post-reconstruction filtering after automatic random and scattering correction.

### Image evaluation

PET data were analyzed by two experienced nuclear medicine specialists (P.F. and C.Z.) on a consensus decision. The readers were blind to the results of ^18^F-FDG PET/CT when reporting Al^18^F‑NOTA‑FAPI-04 PET/CT. Al^18^F‑NOTA‑FAPI-04 tracer uptake was quantified as SUV_max_ (15min/30min/60min), SUV_mean_ (15min/30min/60min), and tumor to background ratio (TBR) from static images 15 min after tracer injection. The TBR is classified as TBR_1_, TBR_2_, and TBR_3_ depending on the background of the mediastinal blood pool, the liver blood pool, and the muscle. △SUV_max-1_ and △SUV_mean-1_ have been defined as the SUV _max-30min_ values minus the SUV _max-15min_ values, and the SUV _mean-30min_ values minus SUV_mean-15min_. △SUV_max-2_ and △SUV_mean-2_ have been defined as the SUV _max-60min_ values minus the SUV _max-30min_ values, and the SUV _mean-60min_ values minus SUV_mean-30min_. ^18^F‑FDG tracer uptake was quantified as SUV_max_ and SUV_mean_. The discrepancies were discussed and a consensus was reached.

### Statistical analysis

Statistical analyses were performed using SPSS software version 23.0 (SPSS, Chicago, IL, USA), GraphPad Prism (version 8.4.2; GraphPad Software, San Diego, Calif), and the *R* language (version 3.6.3, http://www.r-project.org). Quantitative values were expressed as mean ± SD or median and appropriate range, and categorical variables were presented as a rate or percentage. Shapiro–Wilk test for continuous variables shows that all continuous variable data do not meet normal distribution. A comparison of nonparametric data was performed using a Wilcoxon test. For correlation analyses, Kendall's TaU-B test was used to test the correlation between categorical and continuous variables. The Spearman test was used to test the correlation between continuous and non-continuous variables. All statistical tests were performed 2-sided, and *P* < 0.05 indicated statistical significance.

## Results

### Patients’ characteristics

**Fig. 1:**
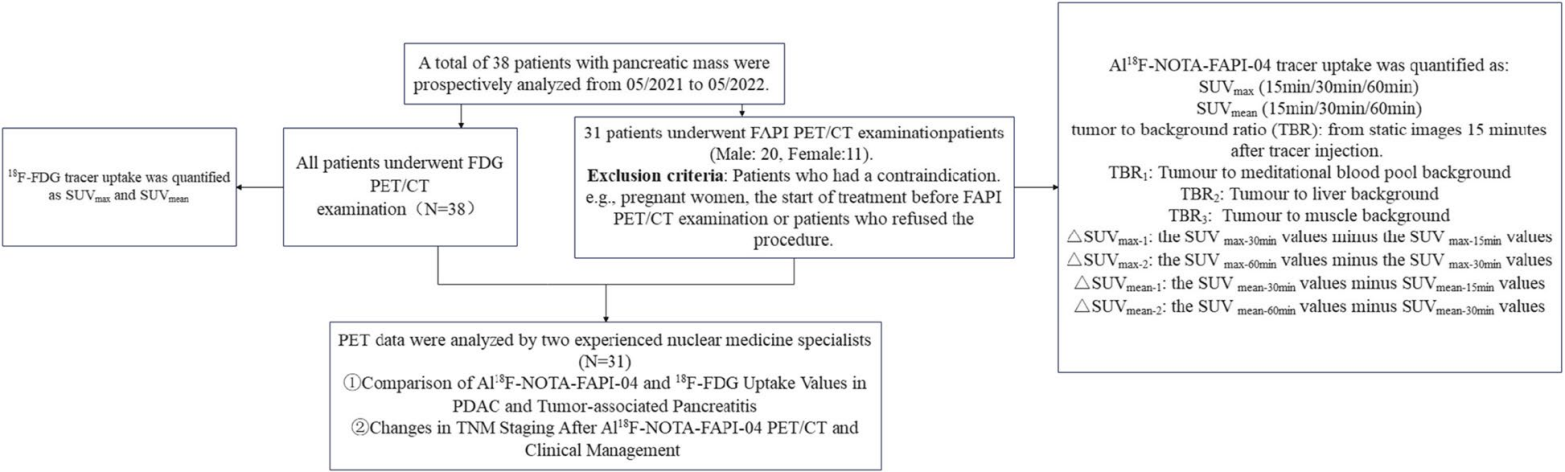
Flow Chart

A total of 31 patients with pancreatic carcinoma (all were adenocarcinoma) have been enrolled and underwent Al^18^F-NOTA-FAPI-04 PET/CT from 05/2021–05/2022, including twenty male and eleven female patients were recruited in the prospective study, with an overall mean age of 58.2 ± 8.5 years at the time of the PET scan (Table 1). The study flow diagram is presented in Fig. 1. The median values of serum CA19-9 and amylase level were 89.2 U/mL (10.6–1000 U/ml, 25^th^-75^th^ percentile: 35.9–144.8 U/mL) and 60.7 U/L (26.3–61089.8 U/L 25^th^-75^th^ percentile 33.8–83.8 U/L), respectively. Among the 31 patients, twelve (38.7%) had jaundice. Most of the patients (*n* = 20, 64.5%) underwent PET/CT examination to clarify the nature of the lesion. Other clinical indications for PET/CT were initial staging (*n* = 9, 29.0%) and restaging (*n* = 2, 6.5%). CE-CT was used in all patients as the most commonly used imaging modality for pancreatic cancer. All cases finally underwent both Al^18^F-NOTA-FAPI-04 and ^18^F-FDG PET/CT.

**Table 1:** Patient Characteristics (*n* = 31 Patients)

Characteristics	Values
Male, n(%)	20 ( 64.5)
Age at FAPI scan, mean ± SD, years	58.2 ± 8.5
Jaundice, n(%)	12 ( 38.7)
Clinical indication for PET/CT	
Clarifying the nature of the lesion, n(%)	20 ( 64.5)
Initial staging, n(%)	9 ( 29.0)
Restaging, n(%)	2 ( 6.5)
Blood Results	
Median (25^th^-75^th^ percentile range) CA19-9^a^, U/ml	89.2 ( 35.9–144.8)
Median (25^th^-75^th^ percentile range) Serum Amylase, U/l	60.7 ( 33.8–83.8)
Location of the primary lesion, n(%)	
Head	16 ( 51.6)
Body	10 ( 32.3)
Tail	5 ( 16.1)
Cholangiectasis observed by Al^18^F-NOTA-FAPI-04 PET/CT, n(%)	14 ( 45.1)
Expansion of the pancreatic duct	22 ( 71.0)

^a^CA19-9 normal range: 0–37 U/mL, ^a^Serum Amylase normal range: 0.01–95 U/L

### Adverse events

All participants tolerated the Al^18^F-NOTA-FAPI-04 PET/CT scan. No Al^18^F-NOTA-FAPI-04-related pharmacological effects or physiological responses occurred. None of the participants reported any abnormal symptoms.

### Biodistribution of Al^18^F-NOTA-FAPI-04-tracers

**Fig. 2:**
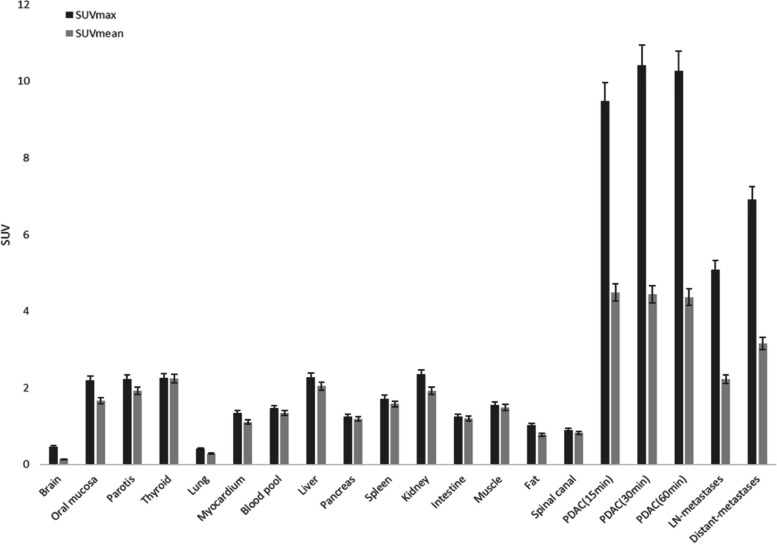
Biodistribution analysis (SUV_max_ and SUV_mean_) of 31 patients with PDAC based on PET/CT imaging 60min after an injection of Al^18^F-NOTA-FAPI-04 tracer molecules (PDAC data included 15 min, 30 min, and 60min after injection)

15min, 30min, and 60min after injection, the value of SUV_max_ and SUV_mean_ for all PDAC were 9.7 ± 2.0 and 4.5 ± 1.2, 10.4 ± 2.3 and 4.5 ± 1.4, 10.3 ± 2.4 and 4.4 ± 1.2, respectively. There was no statistical difference in SUV_max_ and SUV_mean_ values among all these three phases (15min / 30min / 60min). A similar uptake of SUV_max_ and SUV_mean_ values was observed in lymph node metastases (15min: 4.9 ± 1.5 and 2.4 ± 0.9, 30min: 4.9 ± 1.5 and 2.3 ± 0.8, 60min: 5.0 ± 1.6 and 2.3 ± 0.9) and distant metastases (15min: 6.9 ± 2.3 and 3.6 ± 1.8, 30min: 7.1 ± 3.4 and 3.8 ± 1.1, 60min: 7.0 ± 3.0 and 3.6 ± 1.6). There was little Al^18^F-NOTA-FAPI-04 uptake in normal organs, including the uninvolved pancreas, resulting in a high TBR. (e.g., average SUV_max-15min_ tumor/blood pool: 6.9, SUV_max-15min_ tumor/muscle: 6.4, and SUV_max-15min_ tumor/fat: 19.0). A biodistribution of tumor uptake in PDAC manifestations is shown in Fig. 2, along with background activity in normal organs.

### Comparison of Al^18^F-NOTA-FAPI-04 and ^18^F-FDG Uptake Values in PDAC and tumor‑associated pancreatitis

**Fig. 3:**
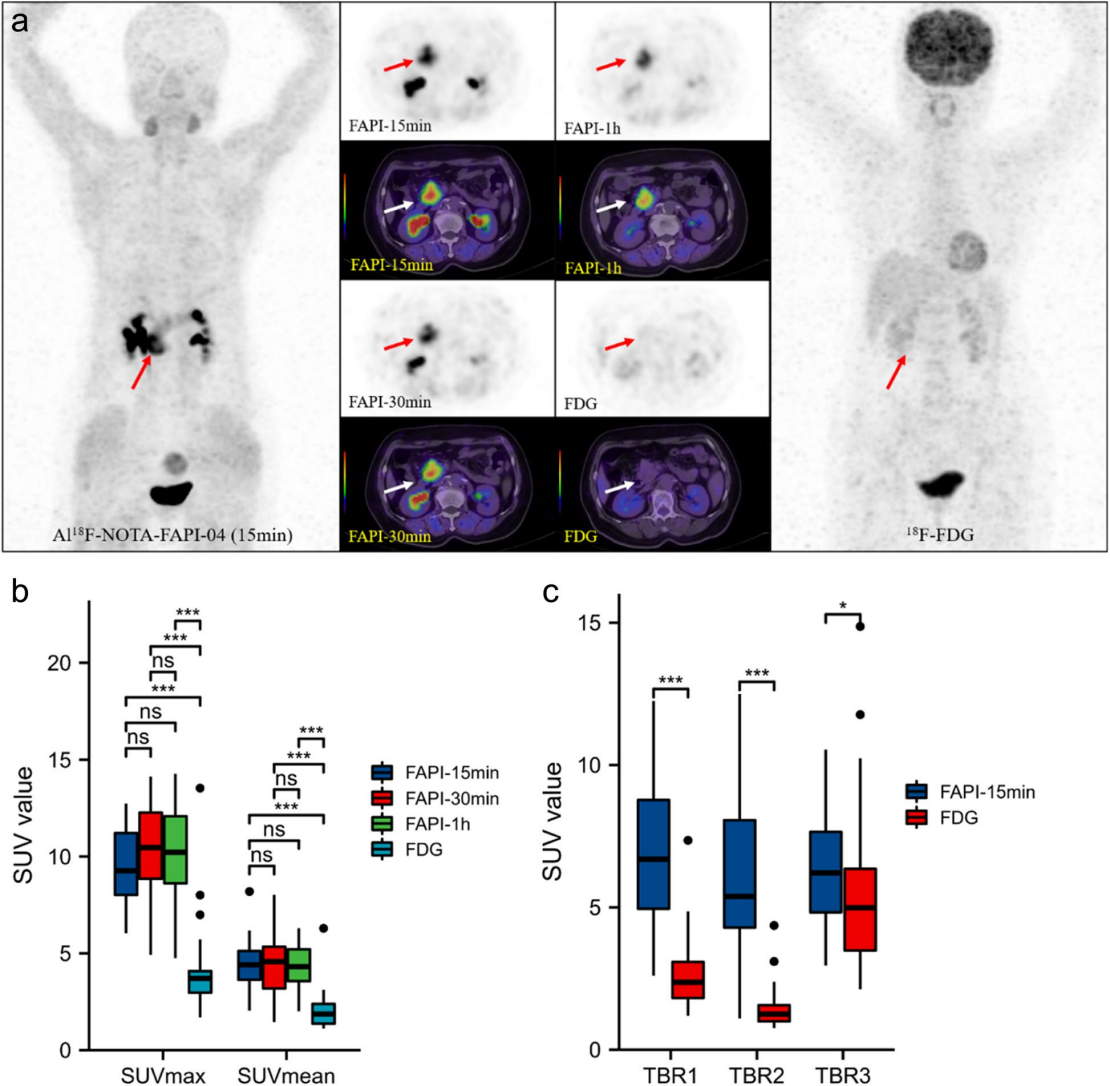
**a**. Female, 62 years old, comparison of Al^18^F-NOTA-FAPI-04 and ^18^F-FDG uptake values in PDAC. Compared with FDG, FAPI PET imaging showed the lesions in the head of the pancreas clearly with higher TBR. The image quality of 15-minute, 30-minute, and 1-hour imaging can satisfy the diagnostic requirements (SUV_max_: 12.9, 12.7, 10.1), where no uptake of FDG showed(SUV_max_: 1.8). **b**. **c**. The SUV_max_, SUV_mean_, TBR_1_, TBR_2_, and TBR_3_ values of Al^18^F-NOTA-FAPI-04 PET / CT imaging were significantly higher than those of FDG

Al^18^F-NOTA-FAPI-04 PET/CT and ^18^F-FDG PET/CT have similar detection abilities for primary pancreatic tumors in this cohort, however, Al^18^F-NOTA-FAPI-04 PET imaging showed a higher radioactive concentration (Fig. 3A). The mean level of SUV_max-15min_, SUV_max-30min,_ and SUV_max-60min_ uptake of PDAC lesions in the Al^18^F-NOTA-FAPI-04 tracer were 9.7 ± 2.0, 10.4 ± 2.3, 10.3 ± 2.4, respectively, and the ^18^F-FDG tracer was 4.1 ± 2.2 (all *P* < 0.01); the mean level of SUV_mean-15min_, SUV_mean-30min_, SUV_mean-60min_ in Al^18^F-NOTA-FAPI-04 tracer were 4.5 ± 1.1, 4.4 ± 1.4, 4.4 ± 1.1, respectively, and the ^18^F-FDG tracer was 2.0 ± 1.0 (all *P* < 0.01) (Fig. 3B). The mean TBR_1_ quantitative measurements of the 15-min Al^18^F-NOTA-FAPI-04 tracer was 6.9 ± 2.5, and that of the ^18^F-FDG tracer was 2.6 ± 1.3 (*P* < 0.01), the mean quantitative measurements of TBR_2_ in 15-min Al^18^F-NOTA-FAPI-04 tracer was 6.2 ± 2.7, and the ^18^F-FDG tracer was 1.5 ± 0.7 (*P* < 0.01), the mean quantitative measurements of TBR_3_ in Al^18^F-NOTA-FAPI-04 tracer was 6.4 ± 1.9, the ^18^F-FDG tracer was 5.4 ± 2.8 (*P* = 0.091) (Fig. 3C).

The presence of 93 lymph nodes was confirmed by lymph node dissection (*n* = 74) or radiographic follow-up (*n* = 19) on 31 patients. The malignant status of 37 lymph nodes was confirmed in 18 cases. By Al^18^F-NOTA-FAPI-04 and ^18^F-FDG PET, a total of 35/37 (94.6%) and 32/37 (86.5%) lymph nodes were shown to be positive, respectively. Sensitivity, specificity, and accuracy for the diagnosis of metastatic lymph nodes were 94.6% (35/37), 80.4% (45/56), and 86.0% (80/93) for Al^18^F-NOTA-FAPI-04 PET/CT and 86.5% (32/37), 71.4% (40/56), and 77.4% (72/93) for ^18^F-FDG PET/CT, respectively.

**Fig. 4:**
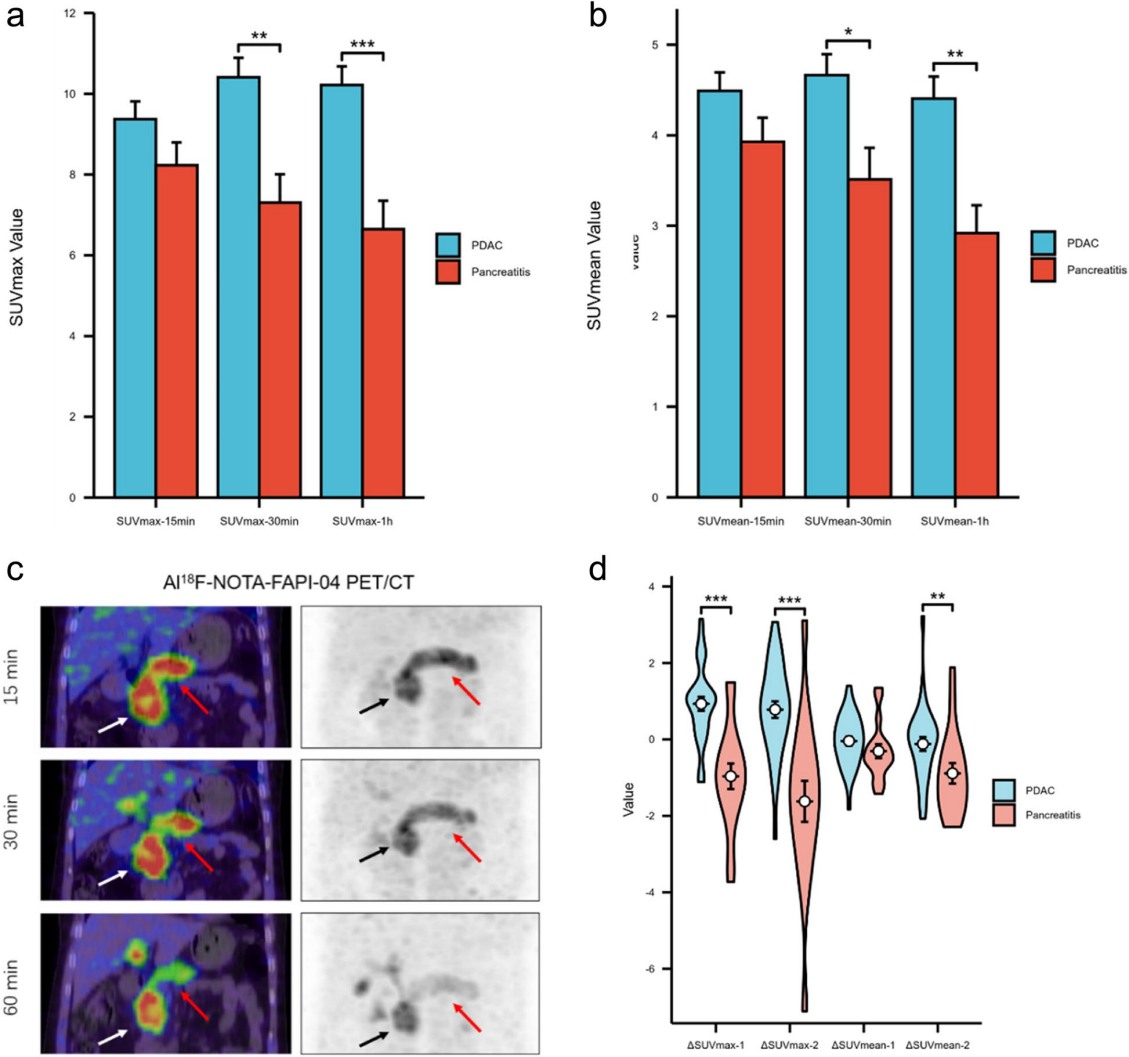
Comparison of Al^18^F-NOTA-FAPI-04 Uptake in PDAC and Tumor-induced Pancreatitis. **a**. The mean level of Al^18^F-NOTA-FAPI-04 uptake values of the PDAC was higher than that of pancreatitis in both SUV_max-30min_ (10.4±2.0 vs. 7.3±2.0, *P *< 0.01) and SUV_max-1h_ (10.2±2.0 vs. 6.6±2.0,* P *< 0.01) subgroups. **b**. The mean level of Al^18^F-NOTA-FAPI-04 uptake values of the PDAC was higher than that of pancreatitis in both SUV_mean-30min_ (4.7±1.0 vs. 3.5±1.0, *P *< 0.05) and SUV_mean-1h_ (4.4±1.0 vs. 2.9±0.9,* P *< 0.01) subgroups. **c**. Female, 71 years old, Al^18^F-NOTA-FAPI-04 PET/CT at 15min, 30min, and 1h showed continued high uptake of PDAC (white and black arrows) located in the head of the pancreas (SUV_max-15min_=10.5, SUV_max-30min_=10.5, SUV_max-15min_=7.7), while pancreatitis (red arrows) showed a decreasing uptake over time (SUV_max-15min_=10.1, SUV_max-30min_=7.4, SUV_max-15min_=4.2). **d.** The violin plot showed that △SUV_max-1_, △SUV_max-2,_ and △SUV_mean-2_ of PDAC and pancreatitis were statistically different

There was a significant increase in tracer uptake in both the PDAC and the rest of the pancreas in 17 out of 31 patients. The semiquantitative analysis of 15min and 30min time point could not distinguish pancreatic tumors from pancreatitis due to similar levels of Al^18^F-NOTA-FAPI-04 uptake. None of these patients had been diagnosed with chronic pancreatitis before Al^18^F-NOTA-FAPI-04 PET/CT imaging. In PDAC, tumor-related exocrine secretion accumulation and consequent pancreatitis are common findings, so our hypothesis is that tumor-associated pancreatitis was likely responsible for the increased uptake of Al^18^F-NOTA-FAPI-04 in most of these patients (Fig. 4C). The pathology following postoperative pathology confirmed that 7/17 patients had pancreatic adenocarcinoma and pancreatitis, following Al^18^F-NOTA-FAPI-04 PET/CT. In all 17 patients, pancreatic Al^18^F-NOTA-FAPI-04 uptake was considered to be a sign of pancreatitis. The mean level of SUV_max-15min_, SUV_max-30min,_ and SUV_max-60min_ uptake in the Al^18^F-NOTA-FAPI-04 tracer were 8.2 ± 1.6, 7.3 ± 2.0, 6.6 ± 2.0, respectively. The mean level of Al^18^F-NOTA-FAPI-04 uptake values of the PDAC was higher than that of pancreatitis in both SUV_max-30min_ (10.4 ± 2.0 vs. 7.3 ± 2.0, *P* < 0.01) and SUV_max-60min_ (10.2 ± 2.0 vs. 6.6 ± 2.0,* P* < 0.01) (Fig. 4A). Similarly, the mean level of Al^18^F-NOTA-FAPI-04 uptake values of the PDAC was higher than that of pancreatitis in both _SUVmean-30min_ (4.7 ± 1.0 vs. 3.5 ± 1.0, *P* < 0.05) and SUV_mean-60min_ (4.4 ± 1.0 vs. 2.9 ± 0.9,* P* < 0.01) (Fig. 4B). The Al^18^F-NOTA-FAPI-04 △SUV_max-1_ (0.9 ± 1.0 vs. -1.0 ± 1.4, *P* < 0.01), △SUV_max-2_ (0.8 ± 1.2 vs. -1.6 ± 2.2, *P* < 0.01), and △SUV_mean-2_ (-0.1 ± 1.0 vs. -0.8 ± 1.1, *P* < 0.01) uptake values of PDAC were higher than the average level of tumor associated pancreatitis (Fig. 4D).

### Changes in TNM staging after Al^18^F-NOTA-FAPI-04 PET/CT and clinical management

**Fig. 5:**
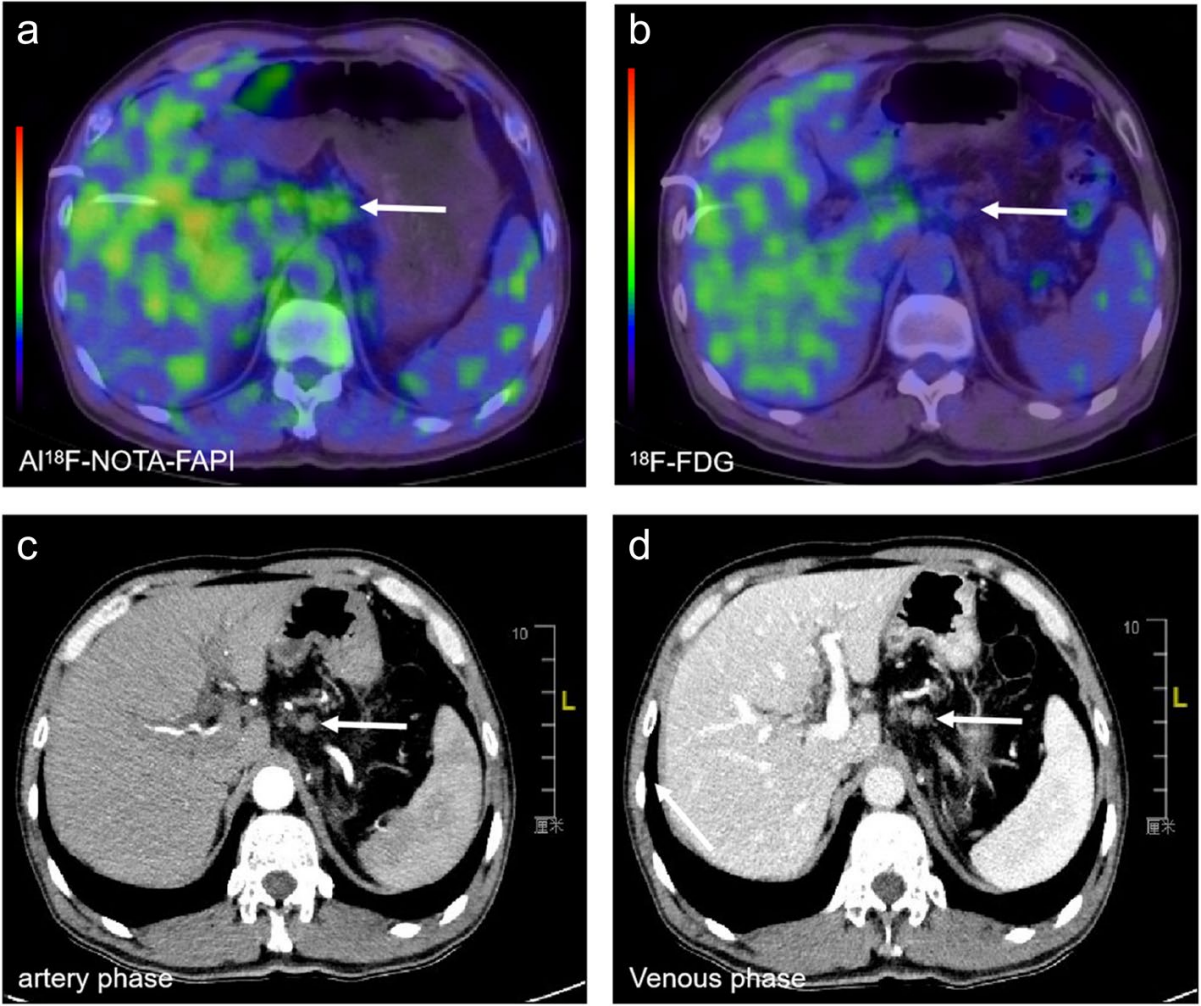
An evaluation of a patient with a local PDAC. Al^18^F-NOTA-FAPI-04 PET/CT showed increased FAPI uptake in lymph nodes (**a**, white arrow, SUV_max-15min_=4.8), which all showed no uptake in ^18^F-FDG PET/CT (**b**, white arrow). Meanwhile, the contrast-enhanced CT showed little enhancement in both arteries (**c**, white arrow) or the venous phase (**d**, white arrow). Thus, the N stage was upstaging to the IIB

Compared to ^18^F-FDG PET/CT, Al^18^F-NOTA-FAPI-04 PET/CT improved the N stage in 16/31 patients (51.6%). A total of 16 patients had their TNM staging upstaged as a result of the new lesions detected by Al^18^F-NOTA-FAPI-04 PET/CT, and all changes are up-staging (Table 2). Seven cases (22.6%) were upstaged by detecting Al^18^F-NOTA-FAPI-04 uptake in abdominal lymph nodes, leading to an upstaging to stage IIB (Fig. 5A-D), and nine cases (29.0%) were upstaged to stage III. Because of newly detected lymph node metastases, the therapeutic regimen was changed in 12 patients (38.7%, surgically resectable to unresectable).

**Table 2:** Comparison of Al^18^F-NOTA-FAPI-04 and ^18^F-FDG PET/CT based TNM staging of 16/31 patients with primary and recurrent/progressive PDAC

No	TNM stage (FAPI‐PET based)	TNM stage (FDG‐PET based)	Additional findings in FAPI‐PET	Staging change
1	T2 N2 M0 Stage3	T2 N1 M0 Stage2B	lymph nodes in abdomen	UP
2	T4 N1 M0 Stage3	T2 N0 M0 Stage1B	a lymph node in abdomen	UP
3	T1 N1 M0 Stage2B	T2 N0 M0 Stage1B	lymph nodes in abdomen	UP
4	T2 N0 M0 Stage1B	T2 N1 M0 Stage2B	lymph nodes in abdomen	UP
5	T1 N0 M0 Stage1A	T1 N0 M0 Stage1A	None	None
6	T3 N0 M1 Stage4	T3 N0 M1 Stage4	None	None
7	T4 N0 M0 Stage3	T4 N0 M0 Stage3	None	None
8	T2 N1 M0 Stage2B	T2 N0 M0 Stage1B	a lymph node in abdomen	UP
9	T1 N0 M0 Stage1A	T1 N0 M0 Stage1A	None	None
10	T3 N0 M0 Stage2A	T3 N0 M0 Stage2A	None	None
11	T1 N0 M0 Stage1A	T1 N0 M0 Stage1A	None	None
12	T1 N1 M0 Stage2B	T1 N0 M0 Stage1A	a lymph node in abdomen	UP
13	T2 N2 M0 Stage3	T2 N2 M0 Stage3	lymph nodes in abdomen	None
14	T2 N1 M0 Stage2B	T2 N0 M0 Stage1B	a lymph node in abdomen	UP
15	T2 N2 M0 Stage3	T2 N1 M0 Stage2B	lymph nodes in abdomen	UP
16	T2 N2 M0 Stage3	T2 N1 M0 Stage2B	lymph nodes in abdomen	UP
17	T2 N2 M0 Stage3	T2 N1 M0 Stage2B	lymph nodes in abdomen	UP
18	T3 N2 M0 Stage3	T3 N1 M0 Stage2B	lymph nodes in abdomen	UP
19	T3 N0 M0 Stage2A	T3 N0 M0 Stage2A	None	None
20	T3 N1 M0 Stage2B	T3 N1 M0 Stage2B	None	None
21	T2 N0 M0 Stage1B	T2 N0 M0 Stage1B	None	None
22	T1 N0 M0 Stage1A	T1 N0 M0 Stage1A	None	None
23	T2 N0 M0 Stage1B	T2 N0 M0 Stage1B	None	None
24	T3 N2 M0 Stage3	T3 N1 M0 Stage2B	lymph nodes in abdomen	UP
25	T3 N0 M0 Stage2A	T3 N0 M0 Stage2A	None	None
26	T2 N1 M0 Stage2B	T2 N0 M0 Stage1B	lymph nodes in abdomen	UP
27	T2 N2 M0 Stage3	T2 N1 M0 Stage2B	lymph nodes in abdomen	UP
28	T2 N1 M0 Stage2B	T2 N1 M0 Stage2B	None	None
29	T2 N1 M0 Stage2B	T2 N1 M0 Stage2B	None	None
30	T2 N1 M0 Stage2B	T2 N0 M0 Stage1B	lymph nodes in abdomen	UP
31	T2 N2 M0 Stage3	T2 N1 M0 Stage2B	lymph nodes in abdomen	UP

### Comparisons of Al^18^F-NOTA-FAPI-04 and ^18^F-FDG between the detection of abnormal uptake in the liver and bones

**Fig. 6:**
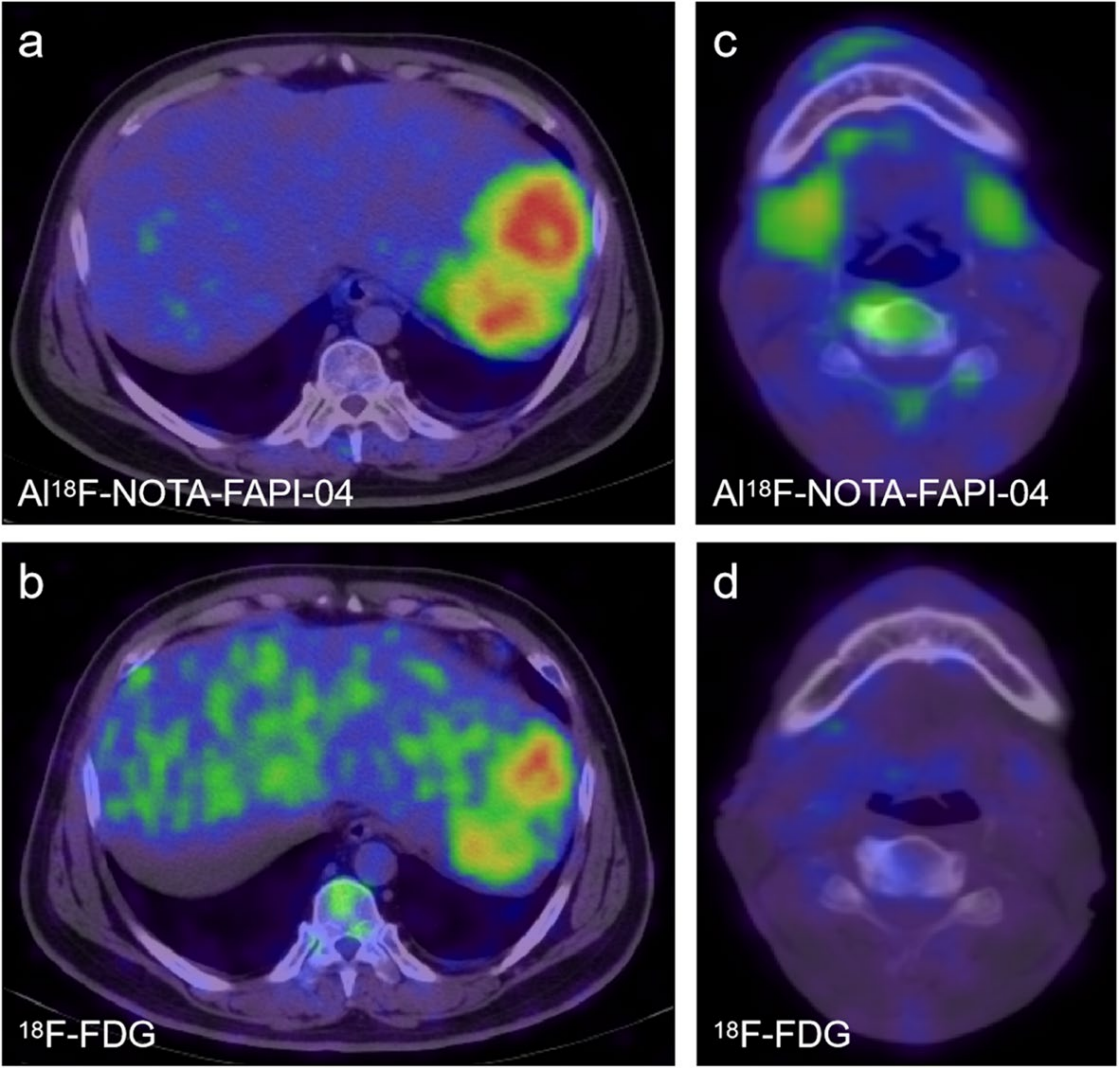
Comparisons between Detection of Extra Abnormal Al^18^F-NOTA-FAPI-04 and ^18^F-FDG Uptake. **a** and **b** both showed Al^18^F-NOTA-FAPI-04 and ^18^F-FDG uptake in the liver metastases (SUV_max_: 10.6 vs. 6.0), demonstrating a better TBR of FAPI than FDG. **c** showed Al^18^F-NOTA-FAPI-04 uptake in cervical bone hyperplasia, while **d** showed no ^18^F-FDG uptake (SUVmax: 3.2 vs. 1.7), suggesting that skeletal lesions should be carefully analyzed

In this cohort, Al^18^F-NOTA-FAPI-04 PET/CT examination found extra uptake in two liver lesions with the SUV_max_ of 10.6, and one bone lesion of the cervical spine with the SUV_max_ of 3.2. While ^18^F-FDG PET/CT examination found two liver lesions with the SUV_max_ of 6.0, no extra ^18^F-FDG uptake was found in the skeleton (Fig. 6 A-D). Two liver lesions were confirmed as liver metastasis by biopsy. Besides, 2 cases of diffused liver fibrosis resulted in overall FAPI uptake in the liver, affecting the interpretation of PET examination. Combined with the imaging of the patient in the previous two years, the lesion in the cervical spine was diagnosed as a benign lesion caused by skeletal degeneration after consultation with two experienced nuclear medicine physicians and two experienced radiologists.

## Discussion

A single-center prospective analysis was conducted to compare Al^18^F-NOTA-FAPI-04 PET/CT and ^18^F-FDG PET/CT for pancreatic lesions diagnosis and malignant tumor staging. PDAC is clinically challenging and is associated with an extremely high mortality rate. ^18^F-FDG PET has proven superior to other anatomical imaging modalities such as CT, MRI, and ultrasound. Improving survival can only be achieved by selecting treatment approaches tailored to the individual patient's condition. In a study comparing FDG PET and FAPI PET/CT in various tumor types, four pancreatic carcinomas showed an upstaging tendency following FAPI PET [12]. Therefore, FAPI-tracer may open new opportunities for staging and restaging PDAC [13].

Our study indicated the following potential advantages for Al^18^F-NOTA-FAPI-04 PET/CT examination: 1) Al^18^F-NOTA-FAPI-04 PET/CT can help diagnose lesions that are difficult to characterize and evaluate on conventional imaging such as CE-CT, ^18^F-FDG PET/CT; 2) Although most studies chose one hour ^68^ Ga-FAPI-04 PET/CT imaging, our study found satisfactory diagnostic efficacy of 15-min and 30-min Al^18^F-NOTA-FAPI-04 PET/CT, which may improve the clinical application scenario and efficiency of FAPI PET/CT; 3) Multi-time point changes in SUV_max_ values in Al^18^F-NOTA-FAPI-04 PET/CT may help to distinguish pancreatic adenocarcinoma foci from tumor-associated pancreatitis; 4) Due to the lower background and higher TBR, Al^18^F-NOTA-FAPI-04 PET/CT imaging is more favorable for showing liver and bone metastases. Thus, the accuracy of TNM staging is further improved.

It is becoming increasingly recognized that CT may be limited in representing pancreatic tumors. There is a correlation between tumor size and sensitivity (83% for lesions less than 2 cm) [14]. Additionally, about 10% of pancreatic adenocarcinomas and metastatic lesions are iso-attenuating on CT and, thus, cannot be detected, even if larger than 2 cm [15]. A CT combined with FDG PET could provide more information than can be obtained by CT alone [16]. Adenocarcinomas of the pancreas typically exhibit an increased FDG uptake area [17]. A pancreatic tumor is characterized by vascular deficiency and abundant desmoplastic stroma, which comprise 90% of its volume. The stroma is composed of extracellular matrix proteins and CAFs [18]. A high TBR and a clear tumor boundary were observed with ^68^ Ga-FAPI PET/CT in pancreatic cancer [19].

Our study showed that the Al^18^F-NOTA-FAPI-04 SUV_max_, SUV_mean_, TBR_1_ and TBR_2_ uptake of pancreatic adenocarcinoma were higher than those of ^18^F-FDG with statistical significance. Abnormal diffused uptake of FAPI uptake in the liver was observed in 2 cases, possibly due to hepatic fibrosis [20, 21]. Therefore, although TBR_3_ was higher than FDG, it did not show statistical significance. This suggests that though the background FAPI of normal liver tissue is low, physicians should pay attention to its sensitivity to liver fibrosis when reviewing Al^18^F‑NOTA‑FAPI-04 PET/CT images. In contrast, choosing the mediastinal blood pool as TBR is less likely to be misinterpreted.

Due to the uptake of FDG in pancreatic adenocarcinoma lesions is not as strong as FAPI, FDG PET imaging often fails to accurately identify the boundary of the lesion, which results in an incorrect measurement of the lesion volume. Our research found that strong uptake of FAPI in displaying pancreatic adenocarcinoma lesions leads to clear boundaries, which may affect the T-staging of the tumor, selection of surgical scope, and delineation of radiotherapy target. Another study also showed how automatic contouring based on FAPIs could improve radiation oncology target definitions for pancreatic cancer [22].

For cases with only pancreatic cancer lesions, a single time point Al^18^F-NOTA-FAPI-04 scan is sufficient for diagnosis. However, 15min time point imaging is not able to differentiate PDAC from tumor-associated inflammation. We recommend multi-time point Al^18^F-NOTA-FAPI-04 scans in the differential diagnosis of PDAC and tumor-associated pancreatitis. This cohort showed statistically significant differences in FAPI SUV_max_ (15min/30min/60min) uptake between PDAC and tumor-associated inflammation. We also found a statistical difference in △SUV_max-1,_ △SUV_max-2_ and △SUV_mean-2_ uptake values in Al^18^F-NOTA-FAPI-04 PET/CT examination between PDAC and tumor-associated inflammation. The tumor-associated inflammation decreased as the uptake time lengthened, while primary pancreatic malignant lesions increased the FAPI uptake value. In other words, even though the pancreas may show diffused FAPI uptake on the same scan, the SUV value at the cancer range is higher than the inflammation. Such changes mentioned above in SUV_max_ values were not observed in ^18^F-FDG PET/CT examination. The SUV_max_ values of ^18^F-FDG were generally low, and the visual assessment might not be quite clear. Al^18^F-NOTA-FAPI-04 PET/CT imaging can be a promising imaging supplement.

Pancreatic adenocarcinoma is staged based on its local and distant spread. Our study showed TNM upstaging caused by Al^18^F-NOTA-FAPI-04 PET/CT imaging over half of the patients (16/31, 51.6%) compared to FDG PET/CT imaging. The most impressive observation was that FAPI PET/CT imaging tended to show a clearer and broader lesion contour. This can affect the assessment of tumor-vessel relationship. However, a CE-CT examination is always recommended if vascular invasions is suspected, regardless of whether FAPI was the racer or FDG. Our study showed that more lymph nodes were involved with Al^18^F-NOTA-FAPI-04 PET/CT than with ^18^F-FDG PET/CT, which would affect N-staging. Sensitivity, specificity, and accuracy for the diagnosis of metastatic lymph nodes for Al^18^F-NOTA-FAPI-04 PET/CT were higher than that of ^18^F-FDG PET/CT. Al^18^F-NOTA-FAPI-04 PET/CT may improve pancreatic adenocarcinoma prognosis by overcoming existing challenges in N staging and rationalizing surgical planning. However, whether FAPI PET/CT imaging can accurately identify lymph node metastasis requires further research on large samples and pathological diagnosis.

A lower liver background has long been an advantage of FAPI PET/CT imaging, revealing more lesions that FDG PET/CT cannot identify. In this cohort, Al^18^F-NOTA-FAPI-04 PET/CT examination found extra uptake in two liver lesions with the SUV_max_ higher than that of ^18^F-FDG. We also found FAPI uptake in the cervical spine in one case. FAPI uptake in bones and joints is a common incidental finding on PET/CT [23]. The FAPI uptake in bone occurs often with degenerative changes where osteophytes are typically visible on CT scans [24]. There have been reports of increased FAPI uptake in cases of bone fractures [25], Schmorl's nodes [25], fibrous dysplasia [26], avascular necrosis [27], mastoiditis [25], bone tuberculosis [28], and myositis ossificans [29] as well. Thus, we sought a discussion with experienced radiologists and nuclear medicine physicians. By comparing the patient's imaging studies over the previous two years, it was concluded that FAPI uptake in this lesion was caused by a local inflammatory response induced by skeletal degeneration. These findings suggest that skeletal FAPI uptake should be carefully interpreted. Comparison with past imaging examination or regular follow-up is necessary and diagnosis valuable.

The equivocal/benign findings which could be present in both scans. Al^18^F-NOTA-FAPI-04 PET/CT may improve the diagnostic rate of PDAC, but it also shows more uptake of benign bone lesions. In addition, FAPI PET can accurately detect more metastatic lymph nodes. In this cohort, FAPI PET did reduce the number of equivocal/benign findings. However, considering the intense uptake of Al^18^F-NOTA-FAPI-04 in liver fibrosis, benign skeletal lesions, etc., we agreed that it is not yet certain that Al^18^F-NOTA-FAPI-04 can replace ^18^F-FDG. Larger sample size, multi-center studies are the focus of future work.

### Limitations

The present study had several limitations. First, this is a single-center study with a small sample size, making it difficult to draw any conclusions about the diagnostic value of the Al^18^F-NOTA-FAPI-04 PET/CT. Second, not all histopathology results were available for lymph node metastasis. There were times when a biopsy by needle or surgery could not be done due to their aggressive nature. Further studies with larger patient cohorts are needed to confirm these results.

## Conclusion

In this prospective study, 15/30-min Al^18^F-NOTA-FAPI-04 PET/CT demonstrated an equivalent detection ability of pancreatic lesions to ^18^F-FDG PET/CT, which may improve the efficiency of clinical application. Multi-time point Al^18^F‑NOTA‑FAPI-04 PET/CT can help differentiate PDAC, tumor-associated inflammation, and inflammatory pancreatic lesions. In terms of TNM staging, Al^18^F-NOTA-FAPI-04 PET/CT performed better than ^18^F-FDG PET/CT.

**List of all quantitative measurements used**

1. **Al** ^18^ **F‑NOTA‑FAPI-04 tracer uptake was quantified as:**
  SUV_max_ (15min/30min/60min)
  SUV_mean_ (15min/30min/60min)
  tumor to background ratio (TBR): from static images 15 minutes after tracer injection.
  TBR_1_: Tumour to meditational blood pool background
  TBR_2_: Tumour to liver background
  TBR_3_: Tumour to muscle background
  △SUV_max-1_: the SUV _max-30min_ values minus the SUV _max-15min_ values
  △SUV_max-2_: the SUV _max-60min_ values minus the SUV _max-30min_ values
  △SUV_mean-1_: the SUV _mean-30min_ values minus SUV_mean-15min_ values
  △SUV_mean-2_: the SUV _mean-60min_ values minus SUV_mean-30min_ values
2. ^**18**^ **F-FDG tracer uptake was quantified as** SUV_max_ and SUV_mean_

## Data Availability

The datasets of current study are available from the corresponding author on reasonable request.

## Abbreviations

CA: Carbohydrate antigen
CAFs: Cancer-associated fibroblasts
CE-CT: Contrast-enhanced CT
FAPI: Fibroblast activation protein inhibitor
MFP: Mass-forming pancreatitis
PDAC: Pancreatic ductal adenocarcinoma
TBR: Tumor-to-background ratio
TNM: Tumor-node-metastasis

## References

[1] Siegel RL, Miller KD, Wagle NS, Jemal A (2023). Cancer statistics, 2023. CA.

[2] Nguyen VX, Nguyen CC, Nguyen BD (2011). 1^8^F-FDG PET/CT imaging of the pancreas: spectrum of diseases. JOP.

[3] Zhang L, Sanagapalli S, Stoita A (2018). Challenges in diagnosis of pancreatic cancer. World J Gastroenterol.

[4] Yeh R, Dercle L, Garg I, Wang ZJ, Hough DM, Goenka AH (2018). The Role of 18F-FDG PET/CT and PET/MRI in Pancreatic Ductal Adenocarcinoma. Abdominal Radiol (New York).

[5] Giesel FL, Kratochwil C, Lindner T (2019). (68)Ga-FAPI PET/CT: Biodistribution and preliminary dosimetry estimate of 2 DOTA-containing FAP-targeting agents in patients with various cancers. J Nuclear Med.

[6] Whittle MC, Hingorani SR (2019). Fibroblasts in pancreatic ductal adenocarcinoma: biological mechanisms and therapeutic targets. Gastroenterology.

[7] von Ahrens D, Bhagat TD, Nagrath D, Maitra A, Verma A (2017). The role of stromal cancer-associated fibroblasts in pancreatic cancer. J Hematol Oncol.

[8] Sun Q, Zhang B, Hu Q (2018). The impact of cancer-associated fibroblasts on major hallmarks of pancreatic cancer. Theranostics.

[9] Zhang Z, Jia G, Pan G (2022). Comparison of the diagnostic efficacy of (68) Ga-FAPI-04 PET/MR and (18)F-FDG PET/CT in patients with pancreatic cancer. Eur J Nucl Med Mol Imaging.

[10] Zhao L, Zhuang Y, Fu K (2020). Usefulness of [(18)F]fluorodeoxyglucose PET/CT for evaluating the PD-L1 status in nasopharyngeal carcinoma. Eur J Nucl Med Mol Imaging.

[11] Wang S, Zhou X, Xu X (2021). Clinical translational evaluation of Al(18)F-NOTA-FAPI for fibroblast activation protein-targeted tumour imaging. Eur J Nucl Med Mol Imaging.

[12] Chen H, Pang Y, Wu J (2020). Comparison of [(68)Ga]Ga-DOTA-FAPI-04 and [(18)F] FDG PET/CT for the diagnosis of primary and metastatic lesions in patients with various types of cancer. Eur J Nucl Med Mol Imaging.

[13] Röhrich M, Naumann P, Giesel FL (2021). Impact of (68)Ga-FAPI PET/CT imaging on the therapeutic management of primary and recurrent pancreatic ductal adenocarcinomas. J Nuclear Med.

[14] Tamm EP, Loyer EM, Faria SC, Evans DB, Wolff RA, Charnsangavej C (2007). Retrospective analysis of dual-phase MDCT and follow-up EUS/EUS-FNA in the diagnosis of pancreatic cancer. Abdom Imaging.

[15] Prokesch RW, Chow LC, Beaulieu CF, Bammer R, Jeffrey RB (2002). Isoattenuating pancreatic adenocarcinoma at multi-detector row CT: secondary signs. Radiology.

[16] Sahani DV, Bonaffini PA, Catalano OA, Guimaraes AR, Blake MA (2012). State-of-the-art PET/CT of the pancreas: current role and emerging indications. Radiographics.

[17] von Schulthess GK, Steinert HC, Hany TF (2006). Integrated PET/CT: current applications and future directions. Radiology.

[18] González-Borja I, Viúdez A, Goñi S (2019). Omics Approaches in pancreatic adenocarcinoma. Cancers.

[19] Pang Y, Zhao L, Shang Q (2022). Positron emission tomography and computed tomography with [(68)Ga]Ga-fibroblast activation protein inhibitors improves tumor detection and staging in patients with pancreatic cancer. Eur J Nucl Med Mol Imaging.

[20] Pirasteh A, Periyasamy S, Meudt JJ (2022). Staging liver fibrosis by fibroblast activation protein inhibitor PET in a human-sized swine model. J Nuclear Med.

[21] Yang AT, Kim YO, Yan XZ (2023). Fibroblast Activation protein activates macrophages and promotes parenchymal liver inflammation and fibrosis. Cell Mol Gastroenterol Hepatol.

[22] Liermann J, Syed M, Ben-Josef E (2021). Impact of FAPI-PET/CT on target volume definition in radiation therapy of locally recurrent pancreatic cancer. Cancers.

[23] Kessler L, Ferdinandus J, Hirmas N (2022). Pitfalls and common findings in (68)Ga-FAPI PET: a pictorial analysis. J Nuclear Med.

[24] Hotta M, Rieger AC, Jafarvand MG (2023). Non-oncologic incidental uptake on FAPI PET/CT imaging. Br J Radiol.

[25] Zheng S, Lin R, Chen S (2021). Characterization of the benign lesions with increased (68)Ga-FAPI-04 uptake in PET/CT. Ann Nucl Med.

[26] Wang Y, Wu J, Liu L, Peng D, Chen Y (2022). 68Ga-FAPI-04 PET/CT imaging for fibrous dysplasia of the bone. Clin Nucl Med.

[27] Liu H, Fu W, Yang X, Chen Y (2022). Increased 68Ga-FAPI uptake in avascular necrosis of femoral head in a patient with nasopharyngeal carcinoma. Clin Nucl Med.

[28] Gong W, Yang X, Mou C, Liu H, Zhang C (2022). Bone tuberculous granulomatous inflammation mimicking malignancy on 68Ga-FAPI PET/CT. Clin Nucl Med.

[29] Gong W, Chen S, He L, Liu W, Zhang C (2022). Intense 68Ga-FAPI uptake in a patient with myositis ossificans: mimicking bone malignancy. Clin Nucl Med.

